# Different decay of antibody response and VOC sensitivity in naïve and previously infected subjects at 15 weeks following vaccination with BNT162b2

**DOI:** 10.1186/s12967-021-03208-3

**Published:** 2022-01-08

**Authors:** Gabriel Siracusano, Alessandra Ruggiero, Zeno Bisoffi, Chiara Piubelli, Luca Dalle Carbonare, Maria Teresa Valenti, Martin Mayora-Neto, Nigel Temperton, Lucia Lopalco, Donato Zipeto

**Affiliations:** 1 Division of Immunology, Transplantation and Infectious Diseases, San Raffaele Scientific Institute, Via Olgettina 58, 20132 Milan, Italy; 2 Department of Neuroscience, Biomedicine and Movement Sciences, University of Verona, Verona, Italy; 3 Department of Infectious, Tropical Diseases and Microbiology, IRCCS Sacro Cuore Don Calabria Hospital Negrar, Verona, Italy; 4 Department of Diagnostic and Public Health, University of Verona, Verona, Italy; 5 Department of Medicine, University of Verona, Verona, Italy; 6 Viral Pseudotype Unit, Medway School of Pharmacy, The Universities of Kent and Greenwich at Medway, Chatham, ME7, 47B UK

**Keywords:** COVID-19, BTN162b2 vaccine, Neutralizing antibodies, SARS-CoV-2 VOCs

## Abstract

**Background:**

COVID-19 vaccines have demonstrated effectiveness in reducing SARS-CoV-2 mild and severe outcomes. In vaccinated subjects with SARS-CoV-2 history, RBD-specific IgG and pseudovirus neutralization titers were rapidly recalled by a single BTN162b2 vaccine dose to higher levels than those in naïve recipients after the second dose, irrespective of waning immunity. In this study, we inspected the long-term kinetic and neutralizing responses of S-specific IgG induced by two administrations of BTN162b2 vaccine in infection-naïve subjects and in subjects previously infected with SARS-CoV-2.

**Methods:**

Twenty-six naïve and 9 previously SARS-CoV-2 infected subjects during the second wave of the pandemic in Italy were enrolled for this study. The two groups had comparable demographic and clinical characteristics. By means of ELISA and pseudotyped-neutralization assays, we investigated the kinetics of developed IgG-RBD and their neutralizing activity against both the ancestral D614G and the SARS-CoV-2 variants of concern emerged later, respectively. The Wilcoxon matched pair signed rank test and the Kruskal–Wallis test with Dunn’s correction for multiple comparison were applied when needed.

**Results:**

Although after 15 weeks from vaccination IgG-RBD dropped in all participants, naïve subjects experienced a more dramatic decline than those with previous SARS-CoV-2 infection. Neutralizing antibodies remained higher in subjects with SARS-CoV-2 history and conferred broad-spectrum protection.

**Conclusions:**

These data suggest that hybrid immunity to SARS-CoV-2 has a relevant impact on the development of IgG-RBD upon vaccination. However, the rapid decay of vaccination-elicited antibodies highlights that the administration of a third dose is expected to boost the response and acquire high levels of cross-neutralizing antibodies.

**Supplementary Information:**

The online version contains supplementary material available at 10.1186/s12967-021-03208-3.

## Background

COVID-19 vaccines have demonstrated effectiveness in reducing SARS-CoV-2 mild and severe outcomes [1, 2]. We have recently demonstrated that in recipients with SARS-CoV-2 history, spike (S)—specific IgG and pseudovirus neutralization titers were rapidly recalled by a single BTN162b2 dose to higher levels than those in naïve recipients after the second dose, irrespective of waning immunity [3]. The long-term duration of the protection is under investigation and growing studies reported a waning immunity over time [4, 5]. As SARS-CoV-2 continually mutates, divergences from the ancestral Wuhan sequence emerged. Along with the B.1.1.7 (Alpha), B.1.351 (Beta), P.1 (Gamma), B.1.1.298 (Delta) variants of concerns (VOCs), the recent B.1.617.2 raises the concern about whether vaccination offers cross-protection between SARS-CoV-2 variants [6]. Here, we inspected the kinetics of IgG-RBD in sera samples from 29 naïve and 10 previously SARS-CoV-2 infected vaccinated subjects over a period of 15 weeks after the first administration. The capacity to provide cross-protection among the VOCs was also investigated.

## Methods

### Vaccinated subjects and serum samples collection

Thirty-five healthcare workers were enrolled for this study. Among them, 26 were naïve and 9 experienced SARS-CoV-2 infections during the second wave of the pandemic in Italy. Infection-naïve subjects had a median age of 37.5 years and the 42% were male. Previously SARS-CoV-2 infected subjects experienced mild symptoms (WHO score 1–2 categorical descriptor) and were mainly male (67%) with a median age of 34 years. Patients’ characteristics are reported in Table 1.

**Table 1:** Patients’ characteristics

	Previously Infected	Infection-naïve	P value
N	9	26	
Age, median year (IQR)	34 (29–57)	37.5 (29–55)	0.6609 (ns)^a^
Gender, M/N (%)	6/9 (67%)	11/26 (42%)	0.2642 (ns)^b^
Time of SARS-CoV-2 infection	2^nd^ wave	n.a	

^a^ non-parametric test Mann–Whitney; ^b^ Fisher exact test

All subjects received two doses of the BTN162b2 vaccine according to the same vaccination schedule as recommended by regulations (21 days between the first and the second dose administration). Serum samples had been collected at four time points: day of first vaccination (W0) and at 3 (W3), six (W6) and fifteen (W15) weeks after the first dose administration. The study was conformed to the principles outlined in the Declaration of Helsinki. Samples were stored in the University of Verona biobank (Ethics Committee approval prot. N. 1538) and in Tropica Biobank of the IRCCS Sacro Cuore Don Calabria Hospital (Ethics Committee approval prot. N. 50,950). All participants signed informed consent.

### SARS-CoV-2 IgG-RBD quantitation

The SARS-CoV-2 IgG II Quant assay (Abbott, Ireland) is a chemiluminescent microparticle immunoassays (CMIA) used for the quantitative measure of IgG-RBD antibodies in human serum. The automated assay was performed according to the manufacturer’s procedure, using the ARCHITECT I System (Abbott). Results were reported as Arbitrary Unit (AU)/mL, according to the following interpretation: AU/mL < 50 = negative, AU/mL > 50 = positive. The lower limit of detection is provided by the manufacturer.

### Cell line

HEK 293 T/17 cells (human embryonic kidney 293 cells) were acquired from National Institute for Biological Standards and Control (NIBSC, UK), cultured in Dulbecco’s Modified Eagle’s Medium supplemented with 4.5 mg/ml glucose, 2 mM L-Glutamine (Lonza), 100 units/mL penicillin–streptomycin (Lonza) and 10% of FBS (Euroclone). Cells were incubated at 37 °C, 5% CO_2_ in humidified atmosphere.

### Production and titration of SARS-CoV-2 pseudotyped particles

SARS-CoV-2 pseudotyped particles were produced in HEK293 T/17 cells co-transfected with the S plasmids, HIV gag-pol and pCSFLW using the FuGENE® HD Transfection Reagent (Promega) according to the manufacturer’s instructions. Supernatant was harvested 72 h later, centrifuged at 500xg for 5 min to clear cell debris and filtered with a 0.45-mm filter. Aliquots were stored at − 80 °C. For titration of pseudoviruses and the neutralization assays, HEK 293 T/17 cells were transfected with pACE2 and pTMPRSS2 for 24 h. Virus infectivity was determined by titration on HEK 293 T/17- ACE2/TMPRSS2 cells as previously described [7].

### Neutralization assays

Neutralization assays were performed by incubating 10^6^ RLU of pseudotyped viruses with endpoint two-fold serial dilutions of heat-inactivated sera samples (56 °C for 30 min) at 37 °C 5%, CO_2_ for 1 h before addition of 10^4^ HEK 293 T/17-ACE2/TMPRSS2 cells per well. After 72 h, the cells were lysed in Luciferase Assay System (Promega) and luciferase activity was measured using a Victor luminometer. Neutralization titers were expressed as ID50 values, defined as the inhibitory dilution at which the half maximal neutralization is achieved. To set up the neutralization assay the International Standard for anti-SARS-CoV-2 antibody (NIBSC code 20/136) and WHO Reference Panel were tested. The panel is composed by five samples ranging from high to low neutralization titer: 20/150 (high titer), 20/148 (mid titer), 20/140 (low titer), 20/142 (negative) (“WHO/BS.2020.2403 Establishment of the WHO International Standard and Reference Panel for anti-SARS-CoV-2 antibody,”) (Additional file 1: Figure S1). A pool of sera from pre-pandemic healthy subjects was used as negative control for each assay as it did not reach the 50% neutralization at the lowest dilution 1/40.

### Statistical analysis

The Wilcoxon matched pair signed rank test was used to assess statistically significant differences between different time points within each vaccinated group. The Kruskal–Wallis test with Dunn’s correction for multiple comparison was applied to compare two groups at fixed times. Graphpad Prism 9 software was used for analysis.

## Results

### Antibody response in naïve and previously infected subjects following vaccination

**Fig. 1:**
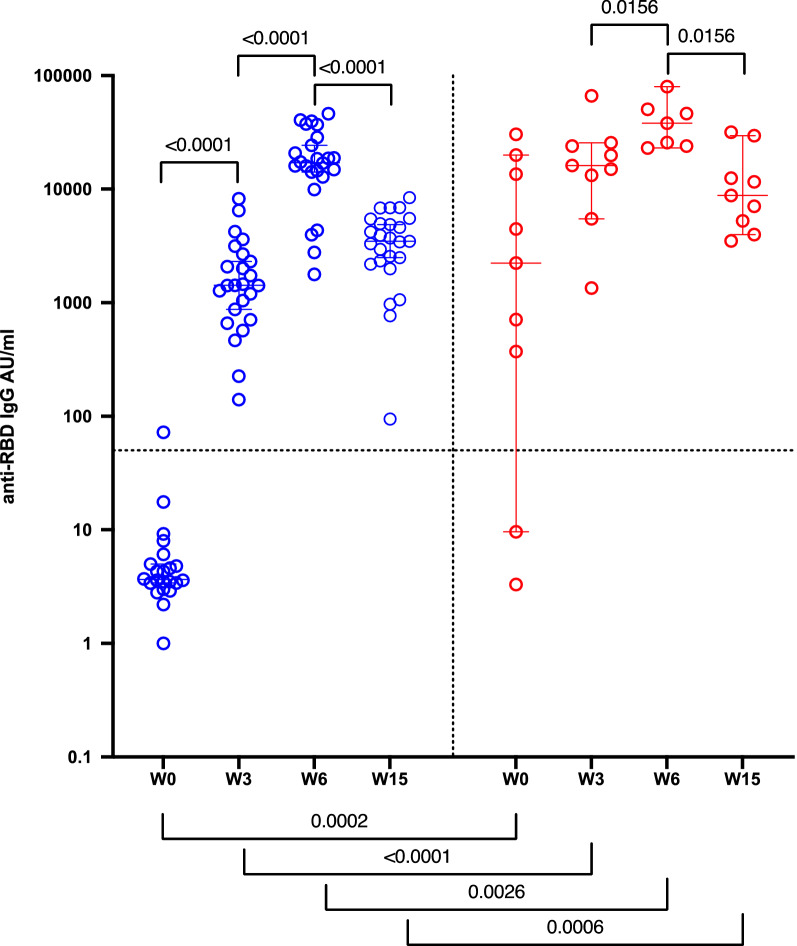
Antibody response in naïve (blue) and previously infected (red) subjects following vaccination. IgG-RBD levels (expressed as AU/ml) in sera from naïve (n = 29) and previously SARS-CoV-2 infected (n = 10) subjects collected at the day of first vaccination (W0), and three (W3), six (W6) and fifteen weeks (W15) from the first vaccination. P˗values were calculated using the Wilcoxon matched-pairs signed ranked test and considered significant if p < 0.05

Antibody (Ab) levels were measured at four time points: day of first vaccination (W0) and at 3 (W3), six (W6) and fifteen (W15) weeks after the first dose administration (Fig. 1). One out of 29 naïve participants had detectable RBD-reactive IgGs before vaccination; conversely, variable levels of IgG-RBD were measured in subjects with history of SARS-CoV-2 infection (median 1471 AU/ml). Consistent with our and other studies [5, 8], the first vaccine dose elicited a more robust IgG-RBD response in previously infected participants compared with naïve (p < 0.0001). Indeed, at W3 from the first injection, naïve subjects had median IgG-RBD levels of 1418 AU/ml, while previously infected individuals raised 15,583 AU/ml. The administration of the second dose in naïve subjects lead to a marked increase of IgG-RBD levels measured at 6 weeks after the first injection, whereas a slight increase was observed in previously infected subjects (median of 17,089 and 38,033 AU/ml, respectively). Although after 15 weeks from vaccination IgG-RBD dropped in all participants (median of 3477 and 8782 AU/ml for naïve and previously infected, respectively), naïve subjects experienced a more dramatic decline (p < 0.0001) than those with previous SARS-CoV-2 infection (p = 0.0156).

### Neutralizing antibody response to SARS-CoV-2 VOCs in naïve and previously infected subjects following vaccination

**Fig. 2:**
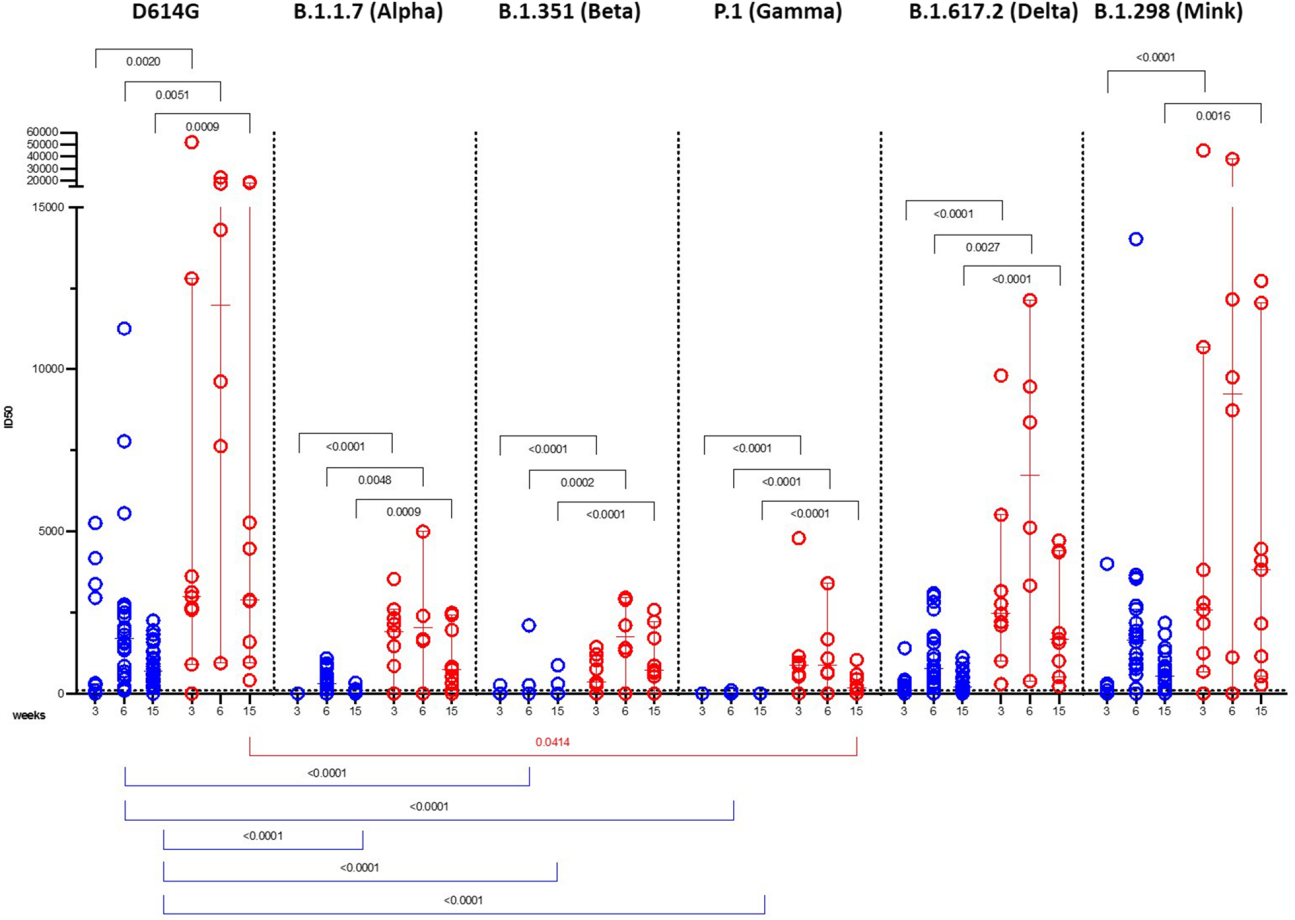
Neutralizing antibody response to SARS-CoV-2 VOCs in infection-naïve (blue) and SARS-CoV-2 previously infected (red) subjects following vaccination. Neutralizing antibody titers (expressed as ID50) against the D614G, B.1.1.7 (Alpha), B.1.351 (Beta), P.1 (Gamma), B.1.617.2 (Delta) and B.1.298 (Mink) SARS-CoV-2 lineages. P˗values were calculated using the Wilcoxon matched-pairs signed ranked test and Kruskal–Wallis test with Dunn’s correction for multiple comparison and considered significant if p < 0.05

The neutralizing potential of post-vaccination sera mirrored those of binding antibodies (Fig. 2). In subjects with prior SARS-CoV-2 infection, the first dose generated neutralizing antibody (nAb) titers to the D614G lineage about 12-fold higher than those raised by naïve participants (p = 0.0020). In particular, 38% naïve subjects had neutralizing antibody titers below the limit of detection at the time of administration of the second vaccine dose (W3), but all developed anti-SARS-CoV-2 nAbs after the second vaccine dose within a broad range of titers (100–11,256 ID50). All subjects with prior SARS-CoV-2 infection had higher neutralizing titers compared with that of naïve subjects after each boost [median ID50 equal to 8962 (p = 0.0051) and 11,962 (p = 0.0009) at W3 and W6, respectively]. As the IgG-RBD titers dropped at W15, median nAb titers against the D614G lineage decreased in both vaccinee’s groups but remained higher in previously infected subjects (median ID50 of 800 for naïve and 2893 for previously infected individuals).

After the first dose, none of the sera from naïve subjects neutralized the B.1.1.7 (Alpha) and P.1 (Gamma) lineages, and 1 out of 23 neutralized the B.1.351 (Beta) lineage. The B.1.1.298 (Mink) and B.1.617.2 (Delta) lineages were more sensitive to neutralization, which correlated with IgG-RBD levels (r = 0.7021, p = 0.0002 and r = 0.4564, p = 0.0286, respectively, see Additional file 2: Table S1).

The B.1.351 (Beta) and P.1 (Gamma) lineages resisted sera neutralization even after the second boost (only 2 and 3 out of 26 sera showed neutralizing activity, respectively). The B.1.1.7 (Alpha) and B.1.617.2 (Delta) lineages were more sensitive to neutralization (with only 7 and 3 out of 24 non-neutralizing sera), along with the B.1.1.298 (Mink), and correlated with IgG-RBD levels (r = 0.7263, p < 0.0001; r = 0.7885, p < 0.0001; r = 0.6682, p = 0.0007, respectively, see Additional file 2: Table S1). Conversely, all the VOCs were neutralized by the sera of previously infected subjects in our study.

The main evidence resulting from our study is the IgG-RBD drop at W15 from the first dose administration. As a consequence of this rapid decay, neutralization against the VOCs decreased in both vaccinee’s groups, although nAb titers remained higher in sera of previously infected subjects, confirming that pre-exposure to SARS-CoV-2 conferred broad-spectrum protection. These findings were confirmed by the strong correlations between binding IgG to RBD and nAb titers (for previously infected.: r = 0.8667, p = 0.0045 for D614G; r = 0.9500, p = 0.0004 for B.1.1.7; r = 0.9205, p = 0.0010; for P.1; r = 0.7833, p = 0172 for B.1.617.2; r = 0.8333, p = 0.0083 for B.1.298; for naïve: r = 0.8506, p < 0.0001 for D614G; r = 0.5155, p = 0.0099 for B.1.1.7; r = 0.6788, p = 0.0003 for B.1.617.2; r = 0.8858, p < 0.0001 for B.1.298, see Additional file 2: Table S1 and Additional file 3: Table S2).

Compared with the D614G lineage, no statistically significant differences were found in the neutralization of B.1.1.7 (Alpha), B.1.351 (Beta), B.1.617.2 (Delta) and B.1.298 (Mink) in previously infected subjects, whereas neutralization ability toward the P.1 lineage was significantly impaired at W15 from the first dose (p = 0.0414). Conversely, in naïve subjects, neutralization was significantly reduced for the B.1.351 and P.1 lineages at W6 and for the B.1.1.7 at W15 (p < 0.0001).

## Discussion

Our study reported that antibodies elicited after two doses of BTN162b2 vaccine rapidly decline after 15 weeks from the first administration, especially in infection-naïve subjects. These subjects developed weaker immunity than those previously exposed to SARS-CoV-2, in terms of serological response and functional neutralization. The expected decline relies on the fact that vaccine induced short-lived plasmablasts that do not necessarily differentiate into long-lived plasma cells [9]. Turner et al.reported that S-binding germinal centre B cells and plasmablasts in draining lymphonodes persist at least 12 weeks after the second dose [10].

Our results are in line with the “hybrid immunity” concept according to which vaccinated COVID˗19 convalescent individuals developed higher immune responses compared to that of naïve or SARS-CoV-2-infected subjects [11]. Similar evidence was reported by the study of Huijskens et al., in which the authors showed that a history of seasonal influenza vaccination has different effects on infection and vaccination response. In vaccinees, the level of antibodies to the homologous strain was reduced in persons with a history of vaccination, whereas the reverse was true for infected persons [12].

More importantly, our data point out the need to monitor the effectiveness of the BTN162b2 vaccine to protect against possible newly emergent VOCs. The B.1.351 and P.1 lineages were more resistant to sera neutralization compared to the B.1.1.7 and B.1.617.2 lineages even after the second boost. The resistance to neutralization of the first two mentioned lineages was also reported for monoclonal antibodies and convalescent plasma, due to the mutations in the NTD and RBD leading to changes in the spike proteins recognized by neutralizing antibodies [13]. Zani et al. reported that the B.1.1.7 lineage (and B.1.525 lineage) bearing the N501Y single mutation in the RBD was robustly neutralized by vaccine-elicited antibodies from naïve subjects, whereas neutralization of the B.351 and P.1 lineages was lower compared to that of the B.1 lineage, although robust [14]. Different from our data, a similar neutralizing response against the B.1, B.1.1.7, and P.1 (and B.1.525) lineages has been reported in BNT162b2-vaccinated healthcare workers, while the B.1.351 and B.1.617.2 lineages showed a consistent partial immune evasion [15]. However, we cannot exclude that this discrepancy might rely on the different neutralization assays employed, i.e. live virus- and, in our case, pseudotyped virus-based assays. Perhaps, differences in spike conformations between live and pseudotyped virus could make the neutralizing epitope differently accessible to neutralizing antibodies. Although variants escape from SARS-CoV-2 humoral immunity has been reported, S-specific CD4 + T-cell activation is not affected by the mutations, at least in the B.1.1.7 and B.1.351 variants [16].

Taken together, our findings suggest that the administration of a third vaccine dose is expected to boost both humoral and cellular response and acquire high levels of cross-neutralizing antibodies as observed in subjects with pre-existing immunity. Therefore, immunological naïve subjects should be prioritised for an additional vaccine administration. Studies reviewed in [17] indicated that one vaccine dose administration may sufficiently protect people who have recovered from COVID-19. However, few epidemiologic studies provided evidence of the benefit of vaccination for previously infected individuals as well. Unvaccinated Kentucky residents who were infected with SARS-CoV-2 in 2020 had significantly higher likelihood of reinfection during May and June 2021 than those who were vaccinated against COVID-19 [18]. The rapid waning of the response elicited by vaccination suggests the need for a potential seasonal vaccine booster, as with the influenza vaccine, in order to be protected from the risk of reinfection and reduce the burden on the healthy system.

A limitation of this study is the number of subjects included in the analysis. Nevertheless, this limitation does not affect data interpretation, since they are consistent with those of other authors [5, 18] and give insight about the effectiveness of BTN162b2 vaccine to confer protection against the current circulating variant B.1.617.2.

## Supplementary Information

**Additional file 1: SARS-CoV-2 % neutralization for the International Standard for anti-SARS-CoV-2 antibody and Reference Panel members**. Normalized percentage neutralization values are plotted against the logarithm of the dilution factors for the International Standard for anti-SARS-CoV-2 antibody (NIBSC code 20/136) and WHO Reference Panel: 20/150 (High titre, H), 20/148 ( Mid titre, M), 20/140 (Low titre, L), 20/142 (negative, N)

**Additional file 2: Table S1. **Correlations between anti-RBD IgG loads and IC50 values for each variant in infection-naïve patients.

**Additional file 3: Table S2.** Correlations between anti-RBD IgG loads and IC50 values for each variant in previously infected patients.

## Data Availability

All data generated or analysed during this study are included in this published article.
