# Supplementary figures and images for: Different decay of antibody response and VOC sensitivity in naïve and previously infected subjects at 15 weeks following vaccination with BNT162b2
Source: J Transl Med. 2022 Jan 8;20:22. doi: 10.1186/s12967-021-03208-3 (PMC8742572; doi:10.1186/s12967-021-03208-3)

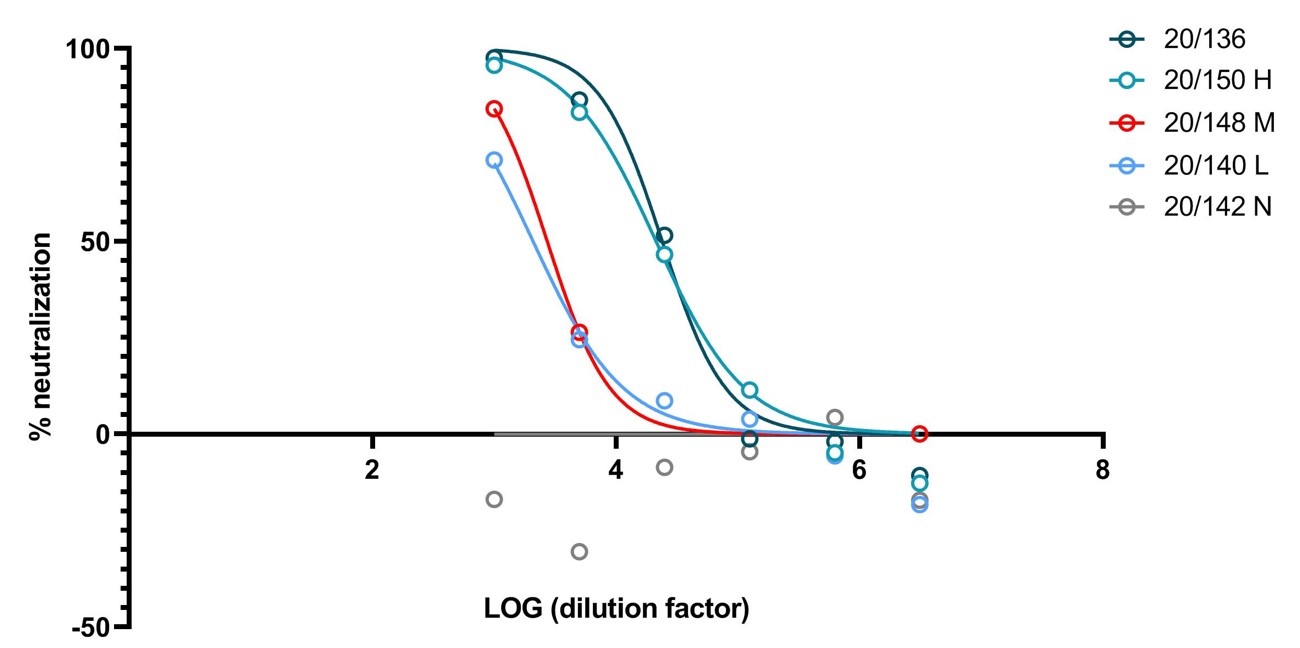

Supplement: Supplementary file 1 — Additional file 1: SARS-CoV-2 % neutralization for the International Standard for anti-SARS-CoV-2 antibody and Reference Panel members. Normalized percentage neutralization values are plotted against the logarithm of the dilution factors for the International Standard for anti-SARS-CoV-2 antibody (NIBSC code 20/136) and WHO Reference Panel: 20/150 (High titre, H), 20/148 ( Mid titre, M), 20/140 (Low titre, L), 20/142 (negative, N) [file 12967_2021_3208_MOESM1_ESM.jpg]
