# Supplementary material for: Different decay of antibody response and VOC sensitivity in naïve and previously infected subjects at 15 weeks following vaccination with BNT162b2
Source: J Transl Med. 2022 Jan 8;20:22. doi: 10.1186/s12967-021-03208-3 (PMC8742572; doi:10.1186/s12967-021-03208-3)
Supplement: Supplementary file 2 — Additional file 2: Table S1. Correlations between anti-RBD IgG loads and IC50 values for each variant in infection-naïve patients. [file 12967_2021_3208_MOESM2_ESM.docx]

| **Infection-naïve** | | D614G | SA | MINK | UK | BR | DELTA |
| --- | --- | --- | --- | --- | --- | --- | --- |
| T1 | rho | 0.05331 | -0.09642 | 0.7021 | IC50 UNV^a^ | IC50 UNV^a^ | 0.4564 |
|  | P (two-tailed) | 0.8091 | 0.6616 | 0.0002 | IC50 UNV^a^ | IC50 UNV^a^ | 0.0286 |
|  | Significant? (alpha = 0.05) | No | No | Yes | na | na | Yes |
|  | Number of XY Pairs | 23 | 23 | 23 | 23 | 23 | 23 |
| T2 | rho | 0.6887 | -0.311 | 0.6682 | 0.7263 | 0.537 | 0.7885 |
|  | P (two-tailed) | 0.0002 | 0.1391 | 0.0007 | <0.0001 | 0.0068 | <0.0001 |
|  | Significant? (alpha = 0.05) | Yes | No | Yes | Yes | Yes | Yes |
|  | Number of XY Pairs | 24 | 24 | 22 | 24 | 24 | 24 |
| T3 | rho | 0.8506 | -0.1824 | 0.8858 | 0.5155 | IC50 UNV^a^ | 0.6788 |
|  | P (two-tailed) | <0.0001 | 0.3936 | <0.0001 | 0.0099 | IC50 UNV^a^ | 0.0003 |
|  | Significant? (alpha = 0.05) | Yes | No | Yes | Yes | na | Yes |
|  | Number of XY Pairs | 24 | 24 | 23 | 24 | 24 | 24 |

**^Table S1. Correlations between anti-RBD IgG loads and ID50 values for each variant in infection-naïve patients^**

^a^ID50 values were uniform across the plate – impossible to perform the correlation. Spearman’s values are shown. UNV=unvaried
