# Supplementary material for: Different decay of antibody response and VOC sensitivity in naïve and previously infected subjects at 15 weeks following vaccination with BNT162b2
Source: J Transl Med. 2022 Jan 8;20:22. doi: 10.1186/s12967-021-03208-3 (PMC8742572; doi:10.1186/s12967-021-03208-3)
Supplement: Supplementary file 3 — Additional file 3: Table S2. Correlations between anti-RBD IgG loads and IC50 values for each variant in previously infected patients. [file 12967_2021_3208_MOESM3_ESM.docx]

**^Table S2. Correlations between anti-RBD IgG loads and ID50 values for each variant in previously infected patients^**

| **Previously infected** | | D614G | SA | MINK | UK | BR | DELTA |
| --- | --- | --- | --- | --- | --- | --- | --- |
| T1 | rho | 0.4833 | 0.7448 | 0.5167 | 0.2929 | 0.5607 | 0.75 |
|  | P (two-tailed) | 0.1938 | 0.0268 | 0.1618 | 0.4408 | 0.1224 | 0.0255 |
|  | Significant? (alpha = 0.05) | No | Yes | No | No | No | Yes |
|  | Number of XY Pairs | 9 | 9 | 9 | 9 | 9 | 9 |
| T2 | rho | 0.9 | 0.8 | 0.6 | 0.8721 | 0.6 | 0.9 |
|  | P (two-tailed) | 0.0833 | 0.1333 | 0.35 | 0.1 | 0.35 | 0.0833 |
|  | Significant? (alpha = 0.05) | No | No | No | No | No | No |
|  | Number of XY Pairs | 5 | 5 | 5 | 5 | 5 | 5 |
| T3 | rho | 0.8667 | 0.6778 | 0.8333 | 0.95 | 0.9205 | 0.7833 |
|  | P (two-tailed) | 0.0045 | 0.0523 | 0.0083 | 0.0004 | 0.001 | 0.0172 |
|  | Significant? (alpha = 0.05) | Yes | No | Yes | Yes | Yes | Yes |
|  | Number of XY Pairs | 9 | 9 | 9 | 9 | 9 | 9 |
